# Armed and accurate: engineering cytotoxic T cells for eradication of leukemia

**DOI:** 10.1186/1472-6750-12-6

**Published:** 2012-02-08

**Authors:** Marko Radic

**Affiliations:** 1 Department of Microbiology, Immunology and Biochemistry, University of Tennessee, Health Sciences Center, Memphis, TN 38163 (USA

## Abstract

Translational medicine depends on a rapid and efficient exchange of results between the bench and the bedside. A recent example from the field of cancer immunotherapy highlights the essential nature of this exchange. Methods have been developed to convert a patient's cytotoxic T cells into efficient and specific killers of cancer cells in patients with leukemia. By using recombinant DNA techniques, a lentiviral vector was constructed to express chimeric antigen receptors in cytotoxic T cells from patients with advanced chronic lymphocytic leukemia. The purpose of the chimeric receptors was to direct the cytotoxic T cell activity against cells causing the cancer. The effect of infusing the engineered T cells back into the cancer patients was tested in a Phase I trial at the University of Pennsylvania, and the initial results were described in two articles from the research team of Dr. Carl June. The remarkable success of this trial should energize further applications of biotechnology in the development of new cancer immunotherapies.

## Main Text

Recent advances in cancer immunotherapy have allowed the conversion of T cells from leukemia patients into efficient and specific killers of their own cancer cells. The new technique has been recently tested with remarkable results. The outward signs of the successful therapy were dramatic and clear. The cancer patients shook with fever, chills and pain; their blood pressure precipitously dropped. They drifted in and out of sleep for days following the infusion of their own engineered killer T cells. The internal struggle peaked between two and three weeks after T cell reinfusion. The load of lysed cancer cells stressed and nearly poisoned the patients' kidneys. Their immune systems, reinvigorated by the immunotherapy, had destroyed nearly 1 kg of cancer cells. No other available treatment could have achieved a comparable therapeutic success. The new treatment, administered by Dr. Carl June and his colleagues of the Abrahamson Cancer Center at the University of Pennsylvania, achieved an unprecedented complete eradication of the cancer in two of the three chronic lymphocytic leukemia (CLL) patients in whom it was tried. It had been a dream of many: the harnessing of the immune system's ferocious power to eliminate an advanced metastatic cancer.

**Figure 1:**
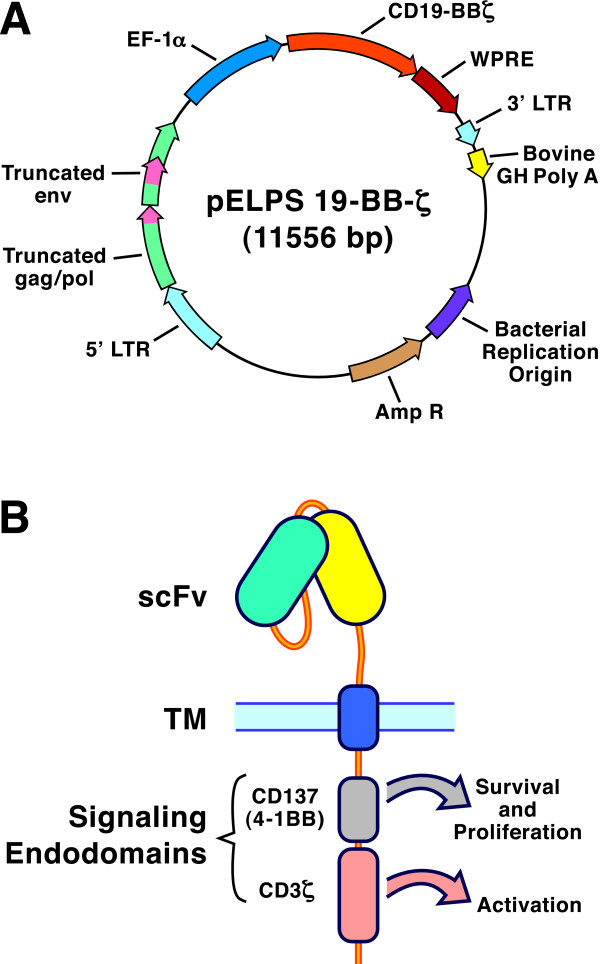
**The biotechnology of chimeric antigen receptors for cytotoxic T cell-based cancer immunotherapy**. A. Map of pELPS 19-BB-3ζ, a lentiviral vector that was approved for clinical trials [1]. The open reading frame encoding the chimeric fusion protein CD19-BBζ contains portions coding for an anti-CD19 single-chain variable region fragment (scFv), the hinge and transmembrane (TM) domain of CD8, and a pair of signaling endodomains (one from CD137, the other from CD3ζ). This fusion protein gene is embedded within a disabled HIV-derived backbone containing truncated gag, pol and env sequences and the 5' and 3' long terminal repeats (LTR). In addition, the construct contains elongation factor 1α (EF-1α) coding sequences, the woodchuck hepatitis virus post-transcriptional regulatory element (WPRE) and the bovine growth hormone polyadenylation (GH poly A) tail, as well as the ampicillin resistance gene and a bacterial replication origin. The total size of the construct is 11,556 base pairs. B. Diagram of the chimeric receptor protein in the T cell plasma membrane. The extracellular antibody recognition domain is linked via the CD8 TM domain to the cytoplasmic signaling domains of CD137 and CD3ζ. These endodomains accomplish the activation of transduced T cells and they stimulate T cell survival and proliferation in vivo.

The stunning results were published in two simultaneous journal articles [1,2], one of the reports presenting data from a single patient [1]. The success was beyond expectation. This, after all, was a Phase I clinical trial, one in which the primary objective was to test different doses of the engineered cytotoxic lymphocytes (CTL). Technically, the success could be traced to the fusion of extracellular and intracellular domains into a new, synthetic receptor for the CD8+ T cells [3]. In immunotherapy jargon, this type of recombinant protein is called a chimeric antigen receptor (CAR). In this case, the extracellular domain was derived from a mouse monoclonal antibody specific for the CD19 surface protein of human B cells. Because CLL is a B cell cancer, CLL cells express CD19. The anti-CD19 antibody was reduced to its smallest active form, a single-chain variable fragment (scFv), and grafted onto a T cell receptor transmembrane domain. On the inside of the cell, the construct was fused to the cytoplasmic signaling domains of CD137 (41BB) and the ζ chain of CD3 (Figure 1). This particular combination of functional domains is credited for achieving the spectacular success of the therapy.

The gene for this CAR was delivered by lentivirus to purified cytotoxic killer T cells from each patient. The HIV-1 virus served as the backbone in the construction of the lentivirus vector. The choice of this virus proved fortunate because the natural host for HIV-1 is a T lymphocyte. Therefore, the vector included sequences that had evolved for replication and expression in T cells. A previous version of this vector was used to treat patients with chronic HIV infections [4]. The CLL patients' purified T cells were transduced in vitro, then stored for the time required to treat each patient with chemotherapy and reduce the numbers of unmodified lymphocytes. This provided the in vivo space to accommodate the engineered T cells.

Once the patients were ready for the experimental treatment, they received between 15 Million and 1 Billion transduced CTL. The CTL rapidly multiplied to approximately 1000-fold higher numbers in vivo [2]. Part of this expansion may be attributed to the presence of target cells. The scientists performing the trial estimated that each engineered CTL destroyed nearly 1000 leukemia cells, until, three weeks after the infusion of the CTL, no detectable cancer cells remained. The peak of CTL proliferation corresponded to the highest level of IFN-γ, IL-6 and CXCL9 in circulation [2]. These cytokines were responsible for the patients' high fever. Once the CLL numbers dropped below detection levels, the CTL numbers normalized and the remaining engineered cells acquired the phenotype of effector and memory T cells. At that time, the patients' overall wellbeing markedly improved.

One side-effect of the therapy is B cell immunodeficiency because the engineered CTL also kill the patients' non-cancerous B cells. This was an anticipated consequence of the therapy because the CTL target antigen, the CD19 receptor, is expressed on all mature B cells and B cell precursors. The transfer of IgG from normal donors (IVIG) can replenish the patients' circulating antibodies and partially compensate for the absence of B cells. The IgG injections will be performed at monthly intervals to reduce the risk of infections in treated patients. Two of the patients were, at the time of the reports, free of cancer recurrence for 6 to 11 months [2]. The third showed a sustained improvement of the CLL.

Various groups of scientists had attempted to modify CTL to attack cancer cells. In pioneering work, Eshhar and collaborators from the Weizmann Institute of Science in Rehovot (Israel) demonstrated that CTL can express a recombinant surface receptor that links an extracellular recognition domain derived from an antibody to an intracellular signaling domain derived from the T cell receptor [5]. Such T cells no longer depend on recognition of HLA-peptide complexes for specific signaling and T cell activation.

Normally, cytotoxic T cells recognize foreign peptides in the groove of an HLA class I receptor. The peptides may be derived from a virus infecting a host cell, which degrades a portion of the newly forming viral proteins into peptides and loads them into a class I HLA. The recognition of the peptides as foreign to the host triggers the T cell to release perforin and granzyme, proteins that form pores in the target cell and initiate a cascade of enzymatic reactions leading to the programmed death of the infected cells. In this manner, the virus is purged from the body.

An analogous reaction is thought to occur between cytotoxic T cells and tumor cells. The oncogenic transformation of a cancer cell induces the expression of proteins that are not expressed in healthy tissues. The immune system can detect the differences in HLA-associated peptides from cancer cells, and the cytotoxic T cells, in consequence, release their cell-degrading effector molecules. However, in advanced cancers, these cytotoxic T cells are depleted and inefficient. Gradually, the cancer cells gain the upper hand and grow to numbers that become more and more difficult to control.

In T cells expressing chimeric antigen receptors, the scFv antibody fragment that binds to antigen substitutes for the TCR binding to HLA plus peptide. The initial success of Gross et al. [5] inspired numerous subsequent studies but obstacles hindered efforts to express recombinant receptors that could bind the desired targets and activate the cytolytic functions of the T cells [6]. Various investigators designed and tested second and third generation CAR fusion proteins because single activation domain CAR showed only limited utility for the stimulation of naïve T cells [6]. The more advanced receptor design incorporated additional endodomains that could simultaneously activate more than one signaling pathway. Experiments by Imai et al. [7] and Finney et al. [8] tested dual activation domains, including the combination of CD3 and CD137 (4-1BB) and found the added activation domains to increase responses of engineered CTL. Subsequently, Milone et al. [3] demonstrated the in vivo efficacy of the new chimeric activation domains in xenogeneic tumor bearing mice. These experiments set the stage for the successful clinical trial at the University of Pennsylvania [1,2].

The breakthrough occurred with the discovery that the cytoplasmic domain from CD137 (4-1BB) enabled strong activation of the engineered T cells, provided that it was inserted between the CD8α transmembrane region and the CD3ζ cytoplasmic domain [3]. This tandem configuration of activating domains merged into a single polypeptide chain the task of delivering two signals needed for the activation of naïve T cells (Figure 1). In this manner, binding of the extracellular antibody domain to antigen could transmit two signals needed to induce the maturation of naïve CD8 T cells into effector T cells. The cells expressing the tandem chimeric antigen receptors received strong signals to proliferate and differentiate into potent cancer-destroying killer T cells [1].

Clearly, the new research will stimulate efforts to extend this approach to other refractory cancers. Still, caveats remain. The numbers of patients treated up to now remain very small and the time elapsed since the treatment is short, thus success could still remain elusive. However, hopes that a promising new therapy is within reach increase with every passing day. In all, more than 25 clinical trials currently explore the use of chimeric T cell receptors in various forms of cancer [9]. It might be feasible to extend the CLL therapy [1,2] to other B lymphocytic neoplasms, as similarly engineered CTL should be capable of attacking any CD19-positive cancer cells. Yet important hurdles remain: It may prove difficult to develop antibodies with exclusive specificity for cancer cells that do not cross-react with healthy tissues. Any cross-reactivity may lead to autoimmunity that could damage vital organs. Furthermore, the violent side-effects associated with the clearance of cancer cells make it desirable to find ways to regulate the rate of CTL activation. For example, there are ways in which a "safety switch" may be incorporated into engineered T cells that could deactivate them in case that the CTL therapy exposes patients to unacceptable complications [10]. The success of the phase I trial with tandem endodomain CARs delivered by lentivirus [1.2] will surely energize the search for additional recombinant immune therapies for advanced cancer.

## Competing interests

The author declares that he has no competing interests.
